# Repurposing ProTAME for Bladder Cancer: A Combined Therapeutic Approach Targeting Cell Migration and MMP Regulation

**DOI:** 10.3390/biology14030263

**Published:** 2025-03-05

**Authors:** Ihsan Nalkiran, Hatice Sevim Nalkiran

**Affiliations:** Department of Medical Biology, Faculty of Medicine, Recep Tayyip Erdogan University, 53020 Rize, Türkiye; ihsan.nalkiran@erdogan.edu.tr

**Keywords:** bladder cancer, proTAME, small molecular inhibitor, MMP2, MMP9, cell migration, molecular docking

## Abstract

Bladder cancer, the fourth most common cancer type among men, remains a therapeutic challenge due to its heterogeneity and frequent development of chemoresistance. Cisplatin-based chemotherapy, often combined with gemcitabine, is the standard treatment, yet resistance and off-target effects in non-cancerous tissues limit its efficacy. This study evaluated the effects of cisplatin, gemcitabine, and the APC/C inhibitor proTAME, both individually and in combination, on cell migration and MMP2/MMP9 expression in RT4 bladder cancer and ARPE-19 normal epithelial cells. Molecular docking analyses were conducted to investigate the interactions of these compounds with MMP2 and MMP9. IC20 values for gemcitabine, cisplatin, and proTAME were applied in scratch-wound healing and quantitative real-time PCR (qRT-PCR) assays. Docking results predicted that proTAME may interact favorably with MMP2 (−9.2 kcal/mol) and MMP9 (−8.7 kcal/mol), showing high computational binding affinities and potential key hydrogen bonds; however, these interactions require further experimental validation. Scratch-wound healing and qRT-PCR assays demonstrated that proTAME-containing combinations were associated with reduced cell migration and decreased MMP2 and MMP9 expression in RT4 cells. Cisplatin combined with proTAME showed the most pronounced reduction in MMP expression and cell migration, with proTAME alone also exhibiting notable inhibitory effects. In ARPE-19 cells, gemcitabine and cisplatin upregulated MMP2 and MMP9 expression, suggesting a potential stress response, whereas proTAME mitigated this effect. These differential effects show the importance of tumor-specific responses in RT4 cells, where proTAME shows promise in enhancing the efficacy of chemotherapy by modulating MMP-related pathways involved in tumor migration and invasion. In conclusion, this study highlights the potential of proTAME as a repurposed agent in bladder cancer treatment due to its association with reduced cell migration and MMP downregulation. While these in vitro and in silico findings suggest a promising role for proTAME in combination therapies, further validation in advanced preclinical models is necessary to assess its therapeutic applicability and safety.

## Simple Summary

Bladder cancer is a common type of cancer, especially among men, and current treatments often become less effective as cancer cells develop resistance. Chemotherapy drugs used today can also cause unwanted side effects by damaging healthy cells. Our study tested a potential drug candidate called proTAME to evaluate if it could help existing chemotherapy drugs better fight bladder cancer without harming normal cells. We found that combining proTAME with standard chemotherapy treatments slowed down the movement of bladder cancer cells, which is important for preventing cancer spread. proTAME also lowered the amounts of certain gene products that cancer cells use to invade healthy tissue. Our results suggest that using proTAME alongside current treatments could make them a more effective treatment option. While these findings are promising, further testing in more advanced models is necessary.

## 1. Introduction

Bladder cancer ranked as the fourth most common cancer type among men in 2023, representing approximately 6% of estimated new cancer cases and 4% of cancer-related deaths [1,2]. Bladder cancer is a complex disease with established risk factors [3]. Cisplatin is of particular interest due to its anticancer activity in various tumors, including solid tumors of the ovary, testes, and head and neck [4]. Cisplatin combination chemotherapy constitutes a fundamental approach in the treatment of many cancers [5]. Gemcitabine, one of the drugs used in combination therapy with cisplatin, is a cytotoxic agent specific to the cell cycle (S-phase) and a deoxycytidine analog that kills cells in the S-phase undergoing DNA synthesis [6]. Compared to gemcitabine alone, cisplatin plus gemcitabine has been associated with a significant survival advantage without adding substantial toxicity [7]. Cisplatin remains a cornerstone in cancer therapy, particularly when combined with agents like gemcitabine, which enhances its efficacy. However, overcoming tumor resistance and minimizing toxicity remain critical hurdles.

A key aspect of cancer progression involves the remodeling of the extracellular matrix, a process largely mediated by matrix metalloproteinases (MMPs) such as MMP2 and MMP9, which facilitate tumor invasion and metastasis. MMPs are enzymes responsible for degrading various protein components of the extracellular matrix [8]. In cancer cells, the MMP family plays a crucial role in tumor invasion and metastasis by damaging the extracellular matrix and basement membrane, activating growth factors, and promoting angiogenesis [9]. Cancer cells rely on the overexpression of MMP2 and MMP9 to migrate from the source cells to adjacent tissues during these processes [10]. Targeting these pathways offers a promising strategy to inhibit cancer spread.

Numerous studies have demonstrated that anaphase-promoting complex/cyclosome (APC/C) and its co-activators play a role in the tumorigenesis of various cancers, revealing their potential as novel therapeutic targets [11,12]. ProTAME, an effective inhibitor of cell cycle progression, blocks the activation of APC/C by the activators CDC20 and CDH1 [13]. Recent research has identified the APC/C and its co-activators as significant contributors to cancer development [14,15]. Inhibiting APC/C activity with agents like proTAME, which blocks its activation via CDC20 and CDH1, opens new avenues for disrupting cancer cell proliferation and invasion. Combining proTAME with conventional chemotherapies such as cisplatin and gemcitabine may therefore represent a novel strategy to enhance treatment outcomes by simultaneously targeting cell cycle regulation and matrix degradation.

This study aims to evaluate the combination effects of cisplatin, gemcitabine, and the APC/C inhibitor proTAME on cell migration as well as MMP2 and MMP9 expression in bladder cancer (RT4 cells) and normal epithelial cells (ARPE-19) as the control. By targeting distinct pathways involved in cancer progression, such as extracellular matrix remodeling and cell cycle regulation, this study seeks to explore the potential therapeutic benefits of these agents when used in combination. A key focus is on proTAME, originally developed to target APC/C activity, to assess its potential for repurposing in bladder cancer treatment. Molecular docking simulations were performed to analyze the binding affinities of these agents to MMP2 and MMP9, alongside scratch assays and gene expression analysis to assess their inhibitory effects on these key enzymes involved in tumor invasion and cell migration. This repurposed approach could offer a novel application for proTAME, expanding its utility beyond cell cycle regulation and positioning it as a candidate for combination therapies to enhance bladder cancer treatment modalities.

## 2. Materials and Methods

### 2.1. Molecular Docking

In this study, the binding affinities of five different ligands to the target proteins MMP2 and MMP9 were evaluated. The compounds used in this study include both therapeutic agents and ligands. The therapeutic agents consist of proTAME (PubChem CID: 56924780), gemcitabine (PubChem CID: 60750), and cisplatin (PubChem CID: 5460033), while I52 (Pub-Chem CID: 5288596, SC-74020) and NFH (PubChem CID: 5287851) are utilized as ligands of MMPs. According to the Protein Data Bank (PDB), the small-molecule ligand I52 is associated with the active site of MMP2 [16], while the co-crystallized ligand NFH (PubChem CID: 5287851) is associated with the active site of MMP9 [17]. These molecules were used as reference molecules for MMP2 and MMP9, respectively. The structures of the therapeutic agents and ligands were retrieved from the PubChem database in SDF format [18]. To prepare the ligands for docking, they were first converted to PDB format using OpenBabel (version 2.4.1) [19] and subsequently subjected to energy minimization using Chimera (version 1.18) [20]. The minimized structures were then converted to PDBQT format using AutoDock Tools 1.5.7 [21], ensuring that the ligands were in their most stable conformation prior to docking.

The 3D crystal structures of the target proteins, MMP2 (PDB, 1HOV) and MMP9 (PDB, 1GKC), were obtained from the PDB Database [22]. Protein preparation involved the removal of water molecules, the addition of polar hydrogens, merging of non-polar hydrogens, and the assignment of Kollman charges using AutoDock Tools 1.5.7 [21]. Binding sites were defined based on co-crystallized ligands or previously reported active site residues within the retrieved structures. Docking simulations were conducted using AutoDock Vina (version 1.1.2) to predict the optimal binding conformations of the ligands within the respective binding sites [21]. Docking calculations were performed using the default exhaustiveness or set to 8. Grid boxes were configured to cover the binding regions of each protein, with the grid dimensions for MMP2 and MMP9 set to X = 40, Y = 40, and Z = 40. The center coordinates (X, Y, Z) of each grid box were positioned based on the respective active site residues (Table 1).

The docking results were ranked based on binding energy values, and the lowest-energy conformations for each protein–ligand complex were selected for further analysis. Visualization of docking poses and protein–ligand interactions was performed using BIOVIA Discovery Studio 2024 Client (Dassault Systèmes, Vélizy-Villacoublay, France). Key interactions, including hydrogen bonds, hydrophobic contacts, and π-based interactions, were identified and compared across the proteins to understand the differential binding mechanisms of the ligands. This approach provided insights into the potential inhibitory effects of the ligands on the target proteins and enabled a comparative analysis of their binding affinities.

### 2.2. Cell Culture and Reagents

ARPE-19 retinal epithelial normal cells kindly provided by Dr. Saliha Eksi, Recep Tayyip Erdogan University and the RT4 human bladder cancer cell line (a recurrent, differentiated transitional papillary bladder tumor, ACC 412) were cultured in RPMI 1640 medium (Gibco, Thermo Fisher, Waltham, MA, USA) supplemented with 10% fetal bovine serum (FBS) (Gibco, Thermo Fisher, Waltham, MA, USA). Cells were maintained at 37 °C in a humidified incubator with 5% CO_2_ and regularly tested for mycoplasma contamination using a PCR-based Mycoplasma PCR Detection Kit (Cat# G238, Viking Way Richmond, BC, Canada). Cells were passaged every 2–3 days using 0.25% trypsin-EDTA (Gibco, Thermo Fisher, Waltham, MA, USA) and were used within 15 passages for all experiments.

Cisplatin (Kocak Pharmaceutical, Istanbul, Turkey), gemcitabine (Kocak Pharmaceutical, Istanbul, Turkey), and proTAME (20 mM stock solution in DMSO, Cat. # I-440, Bio-Techne R&D Systems, Minneapolis, MN, USA) were used in the experiments.

### 2.3. Drug Dose–Response Assays

In a previous study conducted in our laboratory, dose–response curves were constructed for gemcitabine, cisplatin, and proTAME to determine their inhibitory effects on cell viability. For the cytotoxicity assay, we used the MTS assay (CellTiter 96 AQueous MTS Reagent, Promega, Obispo, CA, USA) to determine the IC50 and IC20 values for cisplatin, gemcitabine, and proTAME. RT4 cells were seeded at 1 × 10^4^, and ARPE-19 cells at 2 × 10^4^ cells per well, in 96-well plates with 200 µL of culture medium per well. After overnight incubation, cells were treated with varying concentrations of cisplatin (1–30 µM), gemcitabine (50–5000 nM), ranging from fifty to five thousand nanomolar, and proTAME, (3–36 µM), for 48 h. DMSO controls were included for proTAME-treated groups to account for solvent effects. Following drug exposure, the medium was removed, and 100 µL of fresh culture medium containing MTS reagent (4.2 mg/mL) and phenazine methosulfate (PMS, 0.92 mg/mL, Serva, Heidelberg, Germany) was added to each well and incubated at 37 °C for 3 h. Absorbance was measured at 492 nm using a Multiskan GO microplate reader (Thermo Fisher Scientific, Waltham, MA, USA).

To determine IC20 and IC50 values, cell viability was calculated using the following formula: cell viability, expressed as a percentage, is equal to the absorbance of treated cells divided by the absorbance of control cells, multiplied by one hundred.

Dose–response curves were generated and IC values were determined using the IC50 toolkit (ic50 dot tk) with a nonlinear regression model. Based on three independent biological experiments, each with three technical replicates, we determined the IC20 values as follows: 2.13 µM for cisplatin, 42 nM for gemcitabine, and 12 µM for proTAME [23]. These sub-cytotoxic concentrations were selected to minimize viability effects, allowing us to assess migration and MMP expression changes independently.

### 2.4. Scratch-Wound Healing Assay

The migration of RT4 and ARPE-19 cells was evaluated using a scratch-wound healing assay. The cells were seeded in 24-well plates until reaching ~80% confluency (overnight), at which point a linear scratch was made in the monolayer using a sterile micropipette tip. This was achieved by making two perpendicular linear scratches intersecting at the center of each well. To eliminate any residual cell debris, the cells were washed with Dulbecco’s Phosphate Buffered Saline (DPBS; Gibco, Thermo Fisher Scientific, Waltham, MA, USA) prior to treatment. IC20 doses of the drugs cisplatin, gemcitabine, and proTAME in culture medium supplemented with 10% FBS were added on the cells immediately following the creation of the scratch. To account for potential solvent effects, a DMSO control group was included, in which cells were treated with the same concentration of DMSO as in the proTAME-treated groups for direct comparison. Scratch-wound healing assays were performed in triplicate (*n* = 3 independent replicates), with three technical replicates per condition. Images of the wound area were captured at 0, 24, and 48 h using a digital camera connected to a Leica DM IL LED Inverted Microscope (Leica Microsystems, Wetzlar, Germany) at 4× magnification. Calibration and image preparation were performed in ImageJ 1.54g. The wound area was manually outlined at 0, 24, and 48 h using the ‘Freehand Selection’ tool and measured to determine wound closure over time.

### 2.5. Real-Time qRT-PCR

ARPE-19 and RT4 cells were plated in 6-well plates (3 × 10^5^ cells/well) and allowed to attach overnight. IC20 doses of cisplatin, gemcitabine, proTAME, and their combinations were added on the cells. To mitigate potential solvent effects, a DMSO control group was included, where cells were exposed to the same DMSO concentration as in the proTAME treatment groups for accurate comparison. After incubation with drugs for 48h, the media were removed, and the cells were gently washed three times with cold PBS. Total RNA was isolated using an RNA isolation kit (Macherey-Nagel, Duren, Germany). The High-Capacity cDNA Synthesis Kit (Applied Biosystems, Waltham, MA, USA) was used for cDNA synthesis. A total of 2 µg of RNA was combined with 10 µL of 2X RT master mix, 1 µL of 20X RT enzyme mix, and nuclease-free water to reach a final volume of 20 µL. The mixture was gently mixed and briefly centrifuged. The reaction was incubated in a thermal cycler under the following conditions: 25 °C for 10 min, 37 °C for 120 min, and 85 °C for 5 min, to inactivate the reverse transcriptase. The resulting cDNA was either used directly for downstream applications or stored at −80 °C for later use. Quantitative real-time PCR (qRT-PCR) was conducted in an optical 96-well plate with a Roche LightCycler 480 II, employing the LightCycler 480 Probes Master (Roche, Mannheim, Germany). The primers and hydrolysis probes utilized in the study included TaqMan MMP2 (Hs01548727_m1, Thermo Fisher, Waltham, MA, USA), TaqMan MMP9 (Hs00234579_m1, Thermo Fisher, Waltham, MA, USA), and the housekeeping gene GAPDH, with the forward primer (5′-GAAGGTGAAGGTCGGAGTC-3′), reverse primer (5′-GAAGATGGTGATGGGATTTC-3′), and GAPDH YAK probe (YAK-CAAGCTTCCCGTTCTCAGCCT-BBQ) (TIB MOLBIOL, Berlin, Germany). GAPDH expression levels served as an internal control to normalize the mRNA expression of the target genes. Relative gene expression levels were calculated using the 2^−ΔΔCt^ method. All gene expression experiments were performed with three independent replicates, each consisting of three technical replicates.

### 2.6. Statistical Analysis

Statistics was conducted using the ‘GraphPad QuickCalcs *t*-test Calculator’, available at https://www.graphpad.com/quickcalcs/ttest1.cfm (accessed on 18 January 2025). A *p*-value of less than 0.05 was considered significant. Statistical analyses were performed by comparing each treatment group to either its respective untreated control or DMSO control. The results were expressed as the mean ± standard deviation (SD) from three independent replicates. Gene expression data were analyzed using the ΔCt method, with values normalized to the GAPDH reference gene. The ΔΔCt method was then applied, with comparisons made relative to the ARPE-19 untreated control group. Heatmaps were generated using GraphPad Prism 8.0.1.

## 3. Results

### 3.1. Molecular Docking of ProTAME, Gemcitabine, and Cisplatin with MMP2 and MMP9

**Figure 1:**
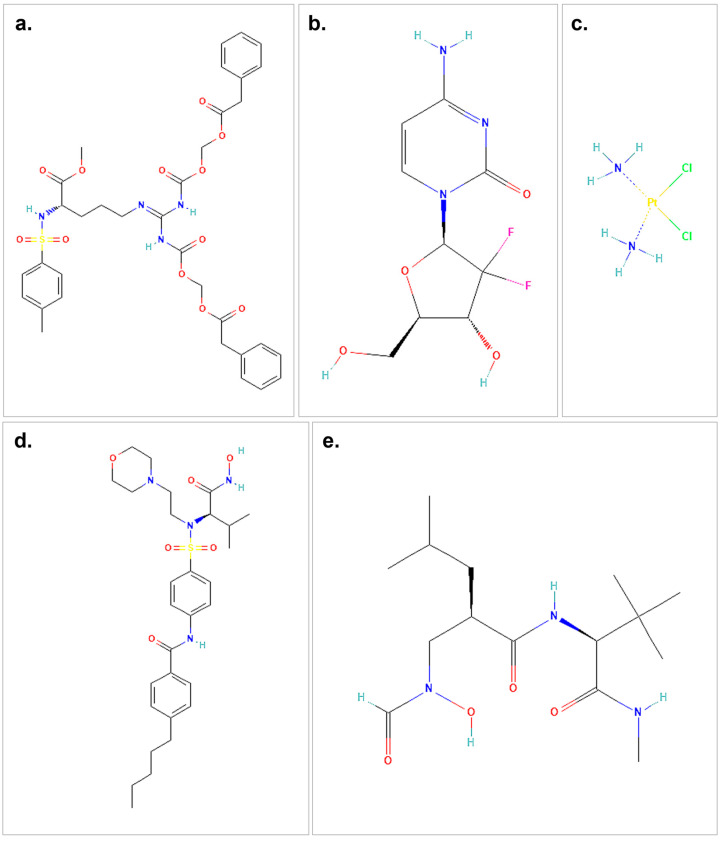
The structures of the therapeutic agents and ligands used in the molecular docking study are shown. (**a**) represents the 2D structure of proTAME. (**b**) illustrates gemcitabine, while (**c**) shows cisplatin. (**d**) presents the small-molecule ligand I52, and (**e**) depicts the 2D structure of the small-molecule ligand NFH. The chemical structures used in this figure were downloaded and modified from the PubChem database, https://pubchem.ncbi.nlm.nih.gov (accessed on 9 October 2024).

The molecular docking analysis revealed significant interactions between the selected compounds (proTAME, gemcitabine, and cisplatin) and the target proteins MMP2 and MMP9 (Figure 1). Docking scores, binding affinities, and the key residues involved in ligand–protein interactions were analyzed to evaluate their potential inhibitory effects. Additionally, the small-molecule ‘I52’ was analyzed as a positive control for MMP2, while ‘NFH’ was used as a positive control for MMP9 in the molecular docking study.

**Figure 2:**
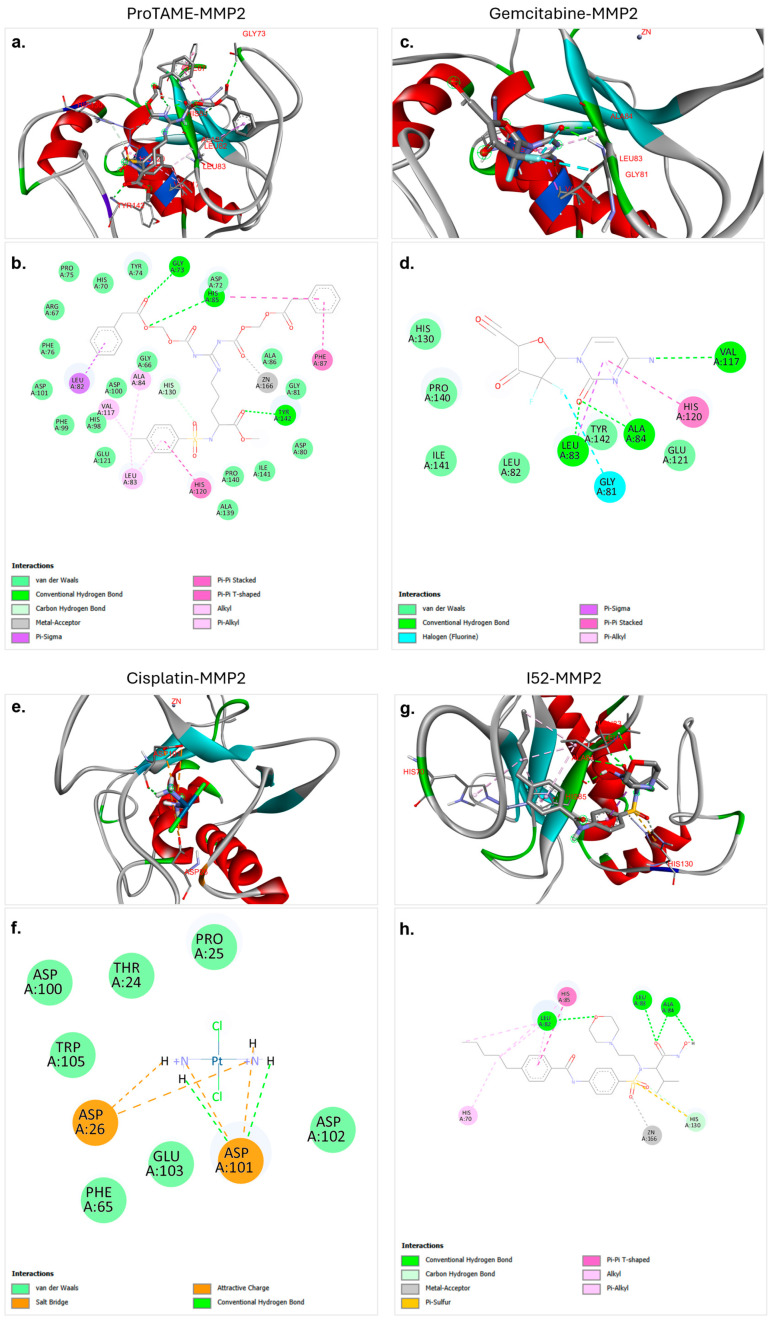
Molecular docking interactions of proTAME, gemcitabine, cisplatin, and I52 with MMP2. (**a**,**b**) ProTAME–MMP2: 3D visualization (**a**) demonstrates stable positioning of proTAME within the active site of MMP2, supported by interactions with key residues, as detailed in the 2D interaction map (**b**). (**c**,**d**) Gemcitabine–MMP2: 3D visualization (**c**) shows gemcitabine effectively bound to active site of MMP2, with the 2D interaction map (**d**) outlining residue-specific interactions. (**e**,**f**) Cisplatin–MMP2: 3D visualization (**e**) illustrates the positioning of cisplatin within the active site of MMP2, with the 2D interaction map (**f**) displaying key stabilizing interactions. (**g**,**h**) I52–MMP2: 3D visualization (**g**) highlights strong binding of I52 to the active site of MMP2, with the 2D interaction map (**h**) summarizing its stabilizing interactions. This figure highlights the varying binding affinities and interaction networks of these ligands within the active site of MMP2.

The molecular docking analysis conducted in this study provided valuable insights into the interactions between MMP2 and four distinct agents: proTAME, gemcitabine, cisplatin, and I52. The results, summarized in Table 2 and shown in Figure 2, highlight significant differences in binding affinities, number of hydrogen bonds, and the structural interactions contributing to stability. Among the agents analyzed, proTAME exhibited the highest binding affinity (−9.2 kcal/mol), closely followed by I52 (−8.1 kcal/mol). Gemcitabine demonstrated moderate binding potential (−7.0 kcal/mol), while cisplatin showed the lowest binding affinity (−3.2 kcal/mol), suggesting varying degrees of effectiveness in targeting MMP2. Hydrogen bonding played a critical role in stabilizing these complexes. ProTAME formed three conventional hydrogen bonds, ensuring strong binding efficiency. I52 formed four hydrogen bonds which demonstrated robust binding affinity. Gemcitabine formed three hydrogen bonds, while cisplatin formed two hydrogen bonds, and both managed to position themselves effectively within the active site of MMP2.

The molecular docking analysis of proTAME with MMP2 revealed significant and strong interactions. The analysis showed that proTAME stably positioned itself within the active site of MMP2, and this binding was primarily reinforced through conventional hydrogen bonds (Figure 2a,b). Conventional hydrogen bonds played a critical role in stabilizing the position of proTAME in the active site and enhancing the stability of the complex. Hydrogen bonds between proTAME and key residues, such as GLY73, HIS85, and TYR142, supported the binding efficiency. Specifically, hydrogen bonds were observed with GLY73 at 2.74 Å, HIS85 at 2.79 Å, and TYR142 at 2.16 Å. In addition to hydrogen bonds, van der Waals forces, carbon–hydrogen bonds, and metal–acceptor and pi-based interactions also played an important role in binding and stability. The presence of these interactions further supports the effective anchoring of proTAME within the active site of MMP2. These findings suggest the potential of proTAME as a selective inhibitor of MMP2, although further studies are necessary to confirm this predicted interaction.

The molecular docking analysis of gemcitabine with MMP2 revealed significant and stable interactions. The analysis demonstrated that gemcitabine effectively positioned itself within the active site of MMP2, with the binding supported by conventional hydrogen bonds, as well as pi-based interactions, halogen bonds, and van der Waals interactions (Figure 2c,d). Conventional hydrogen bonds played a critical role in stabilizing gemcitabine within the active site, significantly contributing to the stability of the ligand–protein complex. Hydrogen bonds were observed between LEU83 and gemcitabine at a distance of 2.02 Å, between ALA84 and gemcitabine at a distance of 2.54 Å, and between VAL117 and gemcitabine at a distance of 3.15 Å. These interactions enhanced the binding efficiency of the ligand and ensured effective positioning within the active site. The presence of these interactions supported the strong binding and stability of gemcitabine in the active site of MMP2. These findings showed the potential of gemcitabine to be evaluated as a potential inhibitor of MMP2. Further experimental validations and computational analyses are required to substantiate these results.

The molecular docking analysis of cisplatin with MMP2 revealed various electrostatic and conventional hydrogen bond interactions that contributed to the stabilization of the complex. Cisplatin was shown to effectively bind within the active site of MMP2, with its binding supported by electrostatic interactions (Figure 2e,f). Notably, conventional hydrogen bonds were found to play a critical role in anchoring cisplatin to the active site. Hydrogen bonds were observed between cisplatin and ASP101 at distances of 2.43 Å and 1.98 Å, and these bonds significantly contributed to the stability of the cisplatin–protein complex. The binding of cisplatin was also facilitated by salt bridges, hydrogen bonds, and van der Waals interactions, further demonstrating its role in stabilizing the ligand within the active site. These findings suggest that despite the lower binding affinity of cisplatin with MMP2, hydrogen bonds, salt bridges, and electrostatic interactions make substantial contributions to the binding stability.

The molecular docking analysis of I52 with MMP2 revealed a strong and favorable interaction. I52, recognized as a ligand of MMP2 in the PDB database, demonstrated effective binding to the active site of MMP2 in our docking analysis, establishing a robust interaction network (Figure 2g,h). The analysis demonstrated that I52 exhibited optimal alignment with the active site residues, contributing to the stabilization of the binding pocket of the protein. Notably, conventional hydrogen bonds played a critical role in this interaction. Hydrogen bonds were observed between LEU82 and I52 at a distance of 2.51 Å, between LEU83 and I52 at a distance of 2.10 Å, and between ALA84 and I52 at distances of 3.02 and 2.14 Å. Additionally, other interactions, including pi-based interactions, metal–acceptor interactions, and carbon hydrogen bonds further enhanced the overall stability and binding affinity of the ligand. These findings support the established role of I52 as a critical ligand for MMP2. Furthermore, this comprehensive interaction profile validates the effectiveness of the docking protocol and provides a reliable benchmark for future molecular docking studies.

The molecular docking analysis conducted in this study also explored the interactions between MMP9 and four distinct ligands: proTAME, gemcitabine, cisplatin, and NFH. The results highlight significant differences in binding characteristics, stabilizing interactions, and hydrogen bond contributions, similar to the findings observed for MMP2 (Table 3). Among these ligands, proTAME exhibited the highest binding affinity (−8.7 kcal/mol), followed by gemcitabine (−8.6 kcal/mol), with NFH demonstrating moderate binding (−6.2 kcal/mol) and cisplatin showing the lowest binding potential (−3.7 kcal/mol). These variations indicate a diverse interaction profile with the active site of MMP9. Hydrogen bonding again played a central role in stabilizing ligand–MMP9 complexes. ProTAME formed three hydrogen bonds, gemcitabine formed one hydrogen bond, NFH formed four hydrogen bonds, and cisplatin formed six hydrogen bonds, highlighting their varying abilities to stabilize within the active site. These findings emphasize the varying potential of these ligands as selective inhibitors of MMP9.

**Figure 3:**
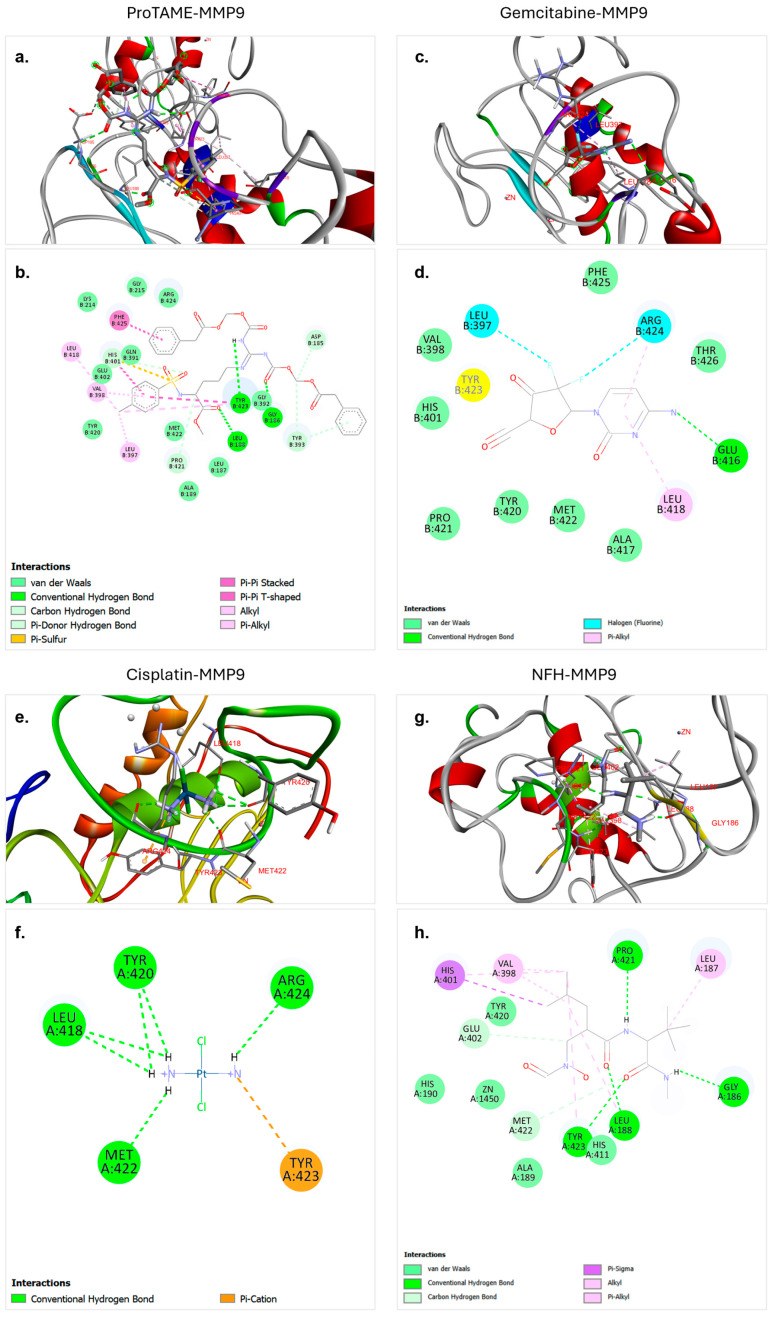
Molecular docking interactions of proTAME, gemcitabine, cisplatin, and NFH with MMP9. (**a**,**b**) ProTAME–MMP9: 3D visualization (**a**) demonstrates the stable binding of proTAME within the active site of MMP9, supported by interactions with surrounding residues. The 2D interaction map (**b**) highlights key stabilizing interactions, including hydrogen bonds and other forces. (**c**,**d**) Gemcitabine–MMP9: 3D visualization (**c**) shows the effective positioning of gemcitabine within the active site of MMP9, with the 2D interaction map (**d**) displaying residue-specific interactions contributing to binding. (**e**,**f**) Cisplatin–MMP9: 3D visualization (**e**) illustrates binding of cisplatin to the active site of MMP9, stabilized by multiple interactions as shown in the 2D interaction map (**f**). (**g**,**h**) NFH–MMP9: 3D visualization (**g**) highlights the positioning of NFH and interactions within the active site of MMP9, with the 2D interaction map (**h**) summarizing key hydrogen bonds and stabilizing forces. This figure emphasizes the varying binding affinities and interaction networks of these ligands with MMP9.

The molecular docking analysis of proTAME with MMP9 revealed strong and favorable interactions (Figure 3a,b). These results indicate that proTAME binds stably and significantly to the active site of MMP9. The binding of proTAME was facilitated by a combination of conventional hydrogen bonds, van der Waals interactions, carbon–hydrogen bonds, and pi-based interactions. Three hydrogen bonds were observed, one with GLY186 at a distance of 3.07 Å and others with LEU188 at a distance of 1.83 Å and TYR423 at a distance of 2.62 Å. The strong predicted binding affinity and diverse stabilizing interactions of proTAME suggest it may have potential as a ligand targeting MM9; nonetheless, experimental validation is essential to confirm its therapeutic applicability.

The molecular docking analysis of gemcitabine with MMP9 revealed strong and stable interactions. These results indicate a significant binding interaction between the ligand and the target protein. According to the analysis, gemcitabine effectively binds to the active site of MMP9, with this binding facilitated by a combination of conventional hydrogen bonds, van der Waals interactions, halogen interactions, and pi-based interactions (Figure 3c,d). A single hydrogen bond was observed between GLU416 and gemcitabine at a distance of 3.21 Å. The strong binding affinity and diverse stabilizing interactions underline the potential of gemcitabine as an effective ligand for therapeutic applications targeting MMP9.

The molecular docking analysis of cisplatin with MMP9 revealed weaker interactions compared to other ligands. However, the analysis demonstrated that cisplatin binds to the active site of MMP9, and this binding is facilitated by a combination of hydrogen bonds and pi–cation interactions (Figure 3e,f). Six hydrogen bonds were observed, including ARG424 at a distance of 2.20 Å, MET422 at a distance of 2.12 Å, LEU418 at a distance of 2.78 Å, TYR420 at distances of 2.86 Å and 2.64 Å, and LEU418 at a distance of 2.88 Å. These multiple hydrogen bonds effectively anchored cisplatin within the active site of MMP9 and significantly contributed to the stability of the complex.

The molecular docking analysis of NFH with MMP9 revealed relatively strong interaction strength. NFH is identified as a ligand in the crystal structure of MMP9 in the PDB and was found to form stable and significant binding interactions with MMP9. The analysis showed that NFH binds effectively to key residues in the active site through hydrogen bonds, carbon–hydrogen bonds, van der Waals forces, and other interactions such as pi-based interactions (Figure 3g,h). Four hydrogen bonds were observed, with LEU188 at 1.83 Å, TYR423 at 2.51 Å, PRO421 at 2.30 Å, and GLY186 at 2.06 Å. These multiple hydrogen bonds contributed significantly to the stability of the NFH–MMP9 complex. The binding affinity and the presence of multiple strong hydrogen bonds highlight the potential of NFH as a ligand targeting MMP9.

### 3.2. Inhibition of Cell Migration by Cisplatin, Gemcitabine, and ProTAME in Scratch-Wound Healing Assay

The scratch-wound healing assay was performed to assess the effects of cisplatin, gemcitabine, proTAME, and their combinations on cell migration in RT4 and ARPE-19. Quantitative measurements and representative images were used to evaluate wound closure, with results highlighting distinct responses between the two cell types.

**Figure 4:**
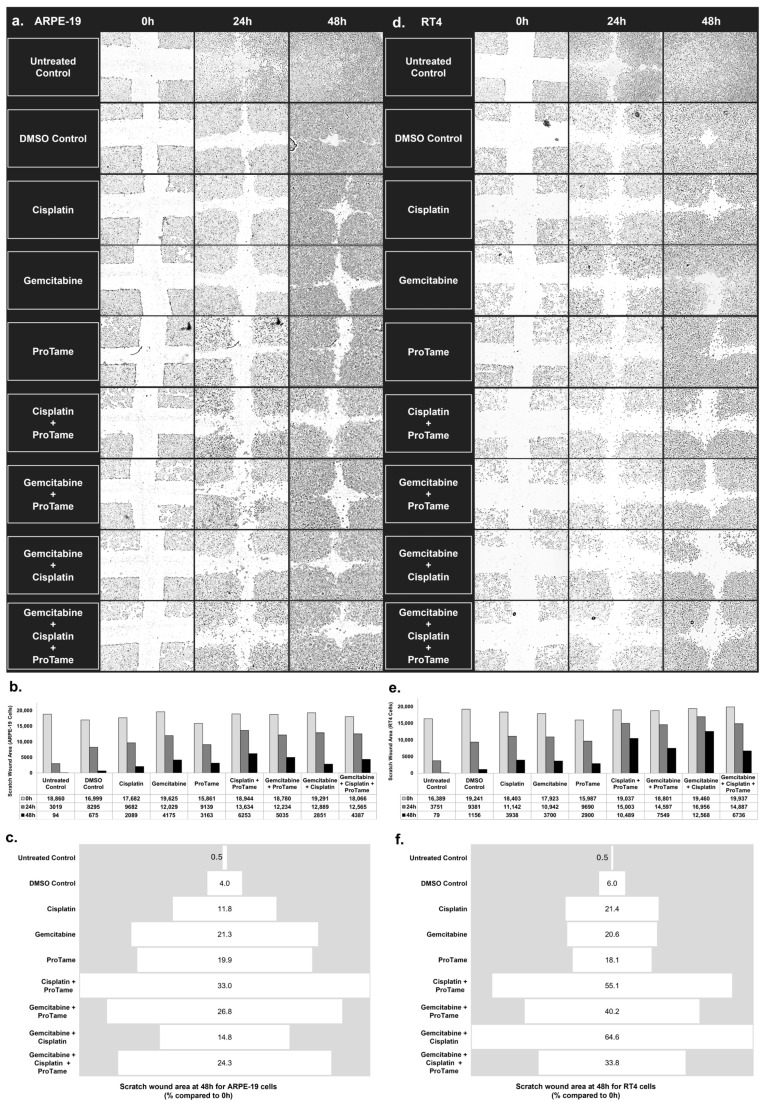
Inhibition of cell migration in ARPE-19 and RT4 cells by cisplatin, gemcitabine, and proTAME treatments in scratch-wound healing assay. (**a**,**d**) The images of scratch-wound healing assays performed on ARPE-19 (**a**) and RT4 (**d**) cells at 0, 24, and 48 h post-treatment. The cells were treated with DMSO control, cisplatin, gemcitabine, proTAME, or combinations of cisplatin+proTAME, gemcitabine+proTAME, gemcitabine+cisplatin, and gemcitabine+cisplatin+proTAME. Images represent one of three independent replicates (*n* = 3), with three technical replicates per condition. Images were captured using an inverted microscope at 4× magnification. (**b**,**e**) Quantification of the scratch-wound area (µm^2^) over time for ARPE-19 (**b**) and RT4 (**e**) cells, with measurements taken at 0, 24, and 48 h. The data show the reduction in scratch area across different treatment groups, indicating varying degrees of cell migration inhibition. (**c**,**f**) The percentage of the scratch-wound area remaining at 48 h relative to the initial wound size (0 h) for ARPE-19 (**c**) and RT4 (**f**) cells.

ARPE-19 cells (Figure 4a) demonstrate progressive wound closure over time in untreated and treated groups. Figure 4b showed that untreated and DMSO-treated controls exhibited significant wound closure, with only 0.5% and 4% of the scratch area remaining at 48 h, respectively (Figure 4c). Among the single-agent treatments, cisplatin and gemcitabine reduced wound closure to 11.8% and 21.3% of the original scratch area, respectively. ProTAME alone resulted in 19.9% of the wound area remaining, indicating a moderate inhibitory effect. Combination therapies showed varied outcomes. Cisplatin+proTAME inhibited wound closure to 33% of the original scratch area, while gemcitabine+proTAME resulted in 26.8% of the wound area remaining at 48 h. The triple-combination of gemcitabine, cisplatin, and proTAME reduced wound closure to 24.3%, indicating a slightly stronger inhibition of migration compared to the doublet combinations. Gemcitabine+cisplatin produced the least effective inhibition of migration among the combinations, leaving 14.8% of the scratch area unclosed.

In RT4 bladder cancer cells, untreated and DMSO-treated controls also showed considerable wound closure over 48 h, with only 0.5% and 6% of the scratch area remaining, respectively (Figure 4d–f). Single-agent treatments demonstrated moderate inhibition of cell migration. Cisplatin and gemcitabine left 21.4% and 20.6% of the wound area unclosed, respectively, while proTAME resulted in 18.1% of the wound area remaining. Combination therapies in RT4 cells showed more substantial inhibitory effects on migration compared to the findings of ARPE-19. Cisplatin+proTAME inhibited wound closure to 55.1% of the original scratch area, demonstrating the strongest inhibition among all treatment groups. Gemcitabine+proTAME reduced the wound area to 40.2%, and the gemcitabine+cisplatin combination resulted in 64.6% wound closure. The triple-agent combination of gemcitabine, cisplatin, and proTAME reduced wound closure to 33.8%.

The results highlight distinct responses between ARPE-19 and RT4 cells. ProTAME-containing combinations, particularly cisplatin+proTAME, demonstrated significant inhibition of cell migration in both cell lines, with the most pronounced effects observed in RT4 bladder cancer cells. These findings suggest that proTAME may enhance the efficacy of chemotherapy in targeting migration pathways, with potential implications for reducing metastatic spread in bladder cancer. These results indicate that proTAME exhibits inhibitory effects on cell migration, yielding outcomes comparable to those observed with cisplatin and gemcitabine treatments.

### 3.3. Modulation of MMP2 and MMP9 Gene Expression by Cisplatin, Gemcitabine, and ProTAME in ARPE-19 and RT4 Cells

**Figure 5:**
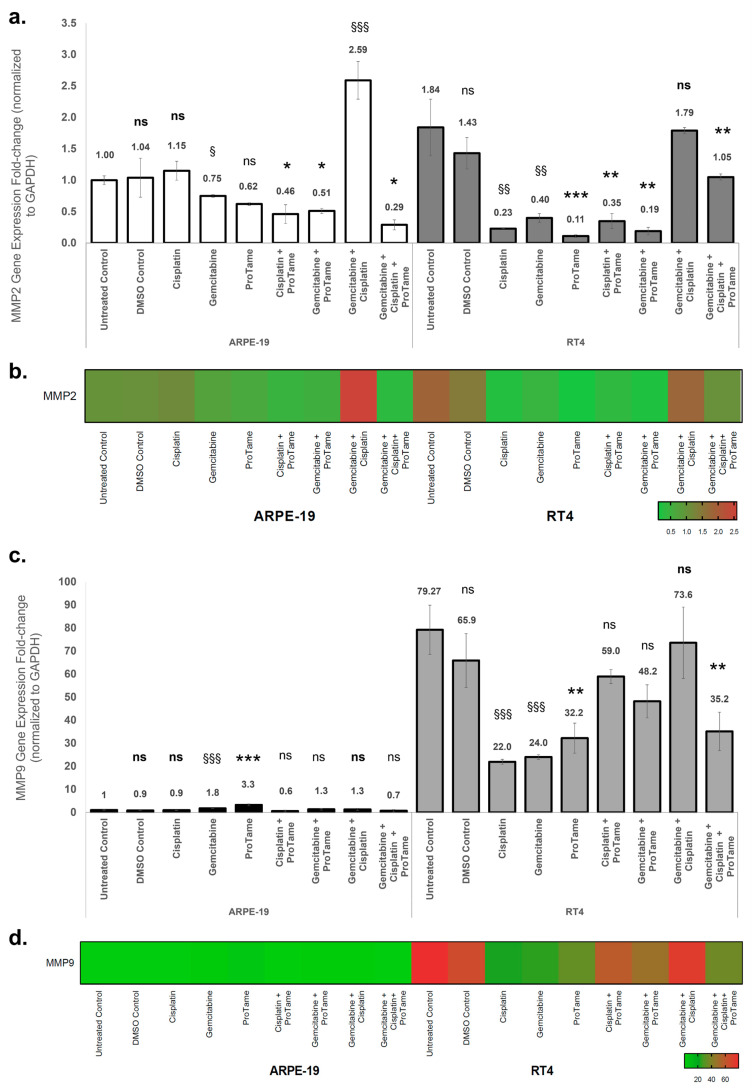
Effects of cisplatin, gemcitabine, and proTAME on MMP2 and MMP9 gene expression in ARPE-19 and RT4 cells. (**a**) MMP2 gene expression fold change in ARPE-19 and RT4 cells following treatment with cisplatin, gemcitabine, proTAME, and their combinations. (**b**) MMP2 gene expression heat map visualization. (**c**) MMP9 gene expression fold change in ARPE-19 and RT4 cells under the same treatment conditions as (**a**). (**d**) MMP9 gene expression heat map visualization. Gene expression levels were normalized to GAPDH, and fold changes were calculated using the 2^−ΔΔCt^ method. The data are presented as fold change relative to the ARPE-19 untreated control, with statistical comparisons conducted against the untreated control for cisplatin-, gemcitabine-, and cisplatin+gemcitabine-treated groups. Statistical significance is indicated as follows: ns: non-significant, *: *p* < 0.05, **: *p* < 0.01, ***: *p* < 0.001. Statistical analyses were performed for proTAME, cisplatin+proTAME, gemcitabine+proTAME, and gemcitabine+cisplatin+proTAME groups compared to DMSO control. The statistical significance is indicated as follows: ns (bold): non-significant, §: *p* < 0.05, §§: *p* < 0.01, §§§: *p* < 0.001. The statistical significance of RT4 untreated control was compared to ARPE-19 untreated control. All experiments were performed with three independent biological replicates, each consisting of three technical replicates, and data are presented as mean ± standard deviation (SD) from three independent experiments.

The effects of cisplatin, gemcitabine, proTAME, and their combinations on MMP2 and MMP9 gene expression in ARPE-19 and RT4 cells were evaluated using qRT-PCR. Gene expression levels were normalized to GAPDH, and fold changes were calculated relative to the ARPE-19 untreated control. In ARPE-19 cells, single-agent treatments with cisplatin and proTAME resulted in non-significant changes in MMP2 expression (Figure 5a). Although the difference was not substantial, gemcitabine treatment alone resulted in a significant downregulation of MMP2 expression (0.62-fold, *p* < 0.05). Combination therapies showed differential effects: cisplatin+proTAME and gemcitabine+proTAME significantly reduced MMP2 expression to 0.46-fold (*p* < 0.05) and 0.51-fold (*p* < 0.05), respectively, whereas gemcitabine+cisplatin induced a marked upregulation of MMP2 expression (2.59-fold, *p* < 0.001). The triple combination of gemcitabine, cisplatin, and proTAME mitigated this effect, resulting in a reduction in MMP2 expression level (0.29-fold, *p* < 0.05), indicating an inhibitory impact. In RT4 cells, cisplatin significantly reduced MMP2 expression to 0.23-fold (*p* < 0.01), while gemcitabine also caused a notable reduction to 0.40-fold (*p* < 0.01) (Figure 5a). ProTAME alone exhibited the most pronounced inhibitory effect, reducing MMP2 expression to 0.11-fold (*p* < 0.001), indicating a strong suppressive effect on MMP2 transcription. Combination therapies further modulated MMP2 expression. The combination of gemcitabine and proTAME resulted in the lowest observed level of expression (0.19-fold, *p* < 0.01), suggesting an enhanced inhibitory effect compared to single-agent treatments with gemcitabine and cisplatin alone. However, the dual combination of cisplatin and gemcitabine did not demonstrate the same level of inhibition, as MMP2 expression increased to 1.79-fold, suggesting a potential treatment-induced, though non-significant, upregulation. Interestingly, the triple combination of cisplatin, gemcitabine, and proTAME resulted in a more moderate reduction in MMP2 expression (1.05-fold, *p* < 0.01) compared to the dual-agent combinations, suggesting a potential counterbalancing effect of proTAME within the combinatory effects of gemcitabine and cisplatin. The heatmap visualization (Figure 5b) demonstrated the differential regulation of MMP2 expression across treatment groups in both cell lines, emphasizing the pronounced inhibitory effects of proTAME-containing therapies in RT4 cells.

In ARPE-19 cells, gemcitabine and proTAME significantly upregulated MMP9 expression by 1.8-fold (*p* < 0.001) and 3.3-fold (*p* < 0.001), respectively, while cisplatin and proTAME showed no significant effects on MMP9 expression (Figure 5c). Combination therapies did not result in significant changes in MMP9 expression. In RT4 cells, untreated controls exhibited a high baseline MMP9 expression level (79-fold relative to ARPE-19 untreated control). Cisplatin significantly reduced MMP9 expression to 22-fold (*p* < 0.001), while gemcitabine resulted in a similar reduction to 24-fold (*p* < 0.001). ProTAME alone further decreased MMP9 expression to 32.2-fold (*p* < 0.01). Among the combinations, the triple-combination therapy demonstrated a notable reduction in MMP9 expression to 35.2-fold (*p* < 0.01), while other-combination therapy groups yielded non-significant decreases in the gene expression. The heatmap visualization (Figure 5d) corroborates the differential modulation of MMP9 expression, with proTAME-containing therapies showing consistent suppression of MMP9 in RT4 cells compared to ARPE-19 cells. These results indicate that proTAME-containing therapies significantly inhibit MMP2 and MMP9 expression, particularly in RT4 bladder cancer cells, suggesting its potential to selectively modulate key pathways involved in tumor invasion and metastasis. Conversely, the upregulation of MMPs in ARPE-19 cells under certain treatment conditions highlights the importance of evaluating off-target effects in normal tissues.

## 4. Discussion

### 4.1. Background and Clinical Relevance

Bladder cancer is one of the most common genitourinary malignancies, with a male predominance of approximately 3:1, making it the fourth most frequent solid malignancy in men. The heterogeneity of bladder cancer complicates the identification of a single reliable biomarker, highlighting the need to expand our understanding of tumorigenesis at the molecular level [24]. Cisplatin-based chemotherapy remains a cornerstone in bladder cancer management; however, it is limited by the emergence of drug resistance mechanisms, including enhanced DNA repair, reduced drug accumulation, and activation of anti-apoptotic signaling pathways. In addition, the associated toxicity in non-cancerous tissues underscores the urgent need for novel therapeutic strategies that enhance efficacy while minimizing adverse effects. The present study investigated the effects of cisplatin, gemcitabine, and proTAME, both individually and in combination, on cell migration and MMP expression in ARPE-19 and RT4 cell lines. Furthermore, molecular docking analyses were performed to assess the binding interactions of these agents with MMP2 and MMP9, offering mechanistic insights into their therapeutic potential.

### 4.2. Effects of Treatments on MMP Expression

In this study, cisplatin and gemcitabine demonstrated distinct effects on MMP expression. In RT4 cells, cisplatin significantly downregulated MMP2 and MMP9 expression, indicating its inhibitory effect on ECM-degrading pathways. Gemcitabine also reduced MMP expression in RT4 cells, though to a lesser extent than cisplatin. ProTAME, an APC/C inhibitor, showed consistent downregulation of MMP2 and MMP9 expression in RT4 cells, suggesting its role in suppressing migration-related pathways. These findings highlight the potential of targeting diverse pathways to improve therapeutic outcomes in bladder cancer, with particular emphasis on inhibiting migration and invasion processes. In contrast, the effects of these agents on ARPE-19 normal epithelial cells were distinct. Gemcitabine and cisplatin combinations led to upregulation of MMP2 and MMP9 expression in ARPE-19 cells, possibly reflecting a stress response associated with tissue remodeling. ProTAME, however, mitigated this upregulation when included in the treatment combinations, underscoring its potential to minimize off-target effects in normal tissues.

Cisplatin is widely used in cancer therapy due to its antitumor activity against various solid tumors, including ovarian, testicular, and head and neck cancers [4]. It remains the gold standard treatment for metastatic urothelial carcinoma [25,26,27,28,29]. Gemcitabine exhibits antitumor activity across various solid tumors, including lung, pancreatic, breast, and bladder cancers [30]. When combined with cisplatin, gemcitabine has shown synergistic effects in several tumor types, such as advanced biliary cancer, without significant additional toxicity [7,31,32,33].

### 4.3. Differential Cellular Responses

The results indicated some differential responses between the cell lines. For instance, the combination of gemcitabine and cisplatin led to a significant increase in MMP2 and MMP9 expression in ARPE-19 cells, which might be due to a stress response to the chemotherapeutic agents even in normal cells. This upregulation of MMPs in ARPE-19 could imply that while these drugs are effective in targeting cancer cells, they may also activate pathways associated with tissue remodeling and repair in normal cells, which could contribute to adverse effects or tissue damage. In contrast, RT4 cells showed a different pattern, with cisplatin alone significantly downregulating MMP2 expression. The combination of cisplatin and gemcitabine in RT4 did not induce the same upregulation of MMPs as observed in ARPE-19, indicating that the cancer cells may have distinct regulatory mechanisms governing MMP expression in response to chemotherapy. These findings demonstrated the complexity of the effect of chemotherapy on different cell types.

The upregulation of MMPs in ARPE-19 cells following gemcitabine and cisplatin combination therapy raises concerns about potential side effects, particularly regarding tissue damage and impaired wound healing in non-cancerous tissues. In this study, the combination of gemcitabine and cisplatin significantly elevated MMP2 and MMP9 expression in ARPE-19 cells, potentially indicating a stress response that activates these enzymes. Given that elevated MMP levels are associated with enhanced tumor invasiveness, this increase could inadvertently promote more aggressive cellular behavior in certain contexts. These findings are consistent with previous studies that demonstrate the critical role of MMPs in facilitating tumor invasion and metastasis by degrading the ECM, activating growth factors, and promoting angiogenesis [9]. Emerging evidence suggests that APC/C and its co-activators (CDC20/CDH1) may influence cancer progression beyond cell cycle regulation, potentially through modulation of EMT and MMP pathways [34]. A previous study demonstrated that disruption of APC/C function using CARP-1 functional mimetics significantly downregulated MMP expression, specifically MMP-10, in medulloblastoma cells, supporting our rationale [35].

### 4.4. Molecular Docking Insights

The molecular docking analyses provided valuable insights into the interactions of cisplatin, gemcitabine, and proTAME with MMP2 and MMP9. ProTAME exhibited the strongest binding affinity to MMP2 and MMP9, supported by multiple hydrogen bonds and hydrophobic interactions, suggesting its potential as a robust inhibitor of these enzymes. Gemcitabine also demonstrated strong binding affinities to both MMP2 and MMP9, mediated by critical hydrogen bonds within the active sites. Cisplatin, while exhibiting lower binding affinities, formed stabilizing electrostatic interactions and hydrogen bonds, contributing to its inhibitory effects on these targets. These results align with the experimental findings, where proTAME-containing combinations showed enhanced inhibition of MMP expression and cell migration, particularly in RT4 cells.

### 4.5. Mechanistic Insights and APC/C Inhibition

ProTAME consistently downregulated MMP2 expression in bladder cancer cells, also exhibiting notable downregulatory effects when used in combination with other agents, which may be linked to the ability of proTAME to block APC/C activation by CDC20 and CDH1, thus interfering with pathways involved in cell cycle progression and protease production [13]. The results indicate that proTAME may counteract the upregulation of MMPs induced by chemotherapeutic agents, thereby reducing the potential for enhanced tumor invasiveness in cancer cells while possibly limiting adverse effects in normal cells. The molecular docking results provide a mechanistic basis for these findings, demonstrating that proTAME and gemcitabine exhibit strong and specific interactions with MMP2 and MMP9, supporting their inhibitory effects observed in experimental assays. These insights highlight the potential of targeting the APC/C pathway in combination with ECM-modulating agents to improve bladder cancer management.

### 4.6. Therapeutic Implications

The inhibitory effects of proTAME on cell migration and MMP expression suggest its therapeutic component in bladder cancer treatment. Previous studies have demonstrated that proTAME effectively overcomes resistance mechanisms linked to APC/C activity in various cancer types, including glioblastoma, ovarian cancer, and diffuse large B-cell lymphoma [36,37,38]. Moreover, our findings indicate that proTAME-containing combination therapies further enhance the inhibition of cell migration in RT4 cells, potentially supporting previous reports of its ability to induce apoptosis and suppress proliferation by targeting APC/C-CDC20 activity [39]. The enhanced inhibition of cell migration observed with proTAME combinations may also be attributed to its ability to modulate apoptotic pathways. Previous research from our laboratory demonstrated that proTAME-containing therapies reduced CDC20 expression and increased the Bax/Bcl-2 ratio in RT4 cells, promoting apoptosis, while reducing this ratio in ARPE-19 cells, indicating a selective effect on cancer cells [23].

### 4.7. Limitations and Future Directions

The differential effects of proTAME and chemotherapeutic combinations on MMP expression and cell migration observed in this study highlight the potential benefits of targeting multiple pathways in bladder cancer treatment. The addition of proTAME to chemotherapy regimens could be suggested to help mitigate MMP-mediated invasion and improve therapeutic outcomes by preventing metastatic progression. This study specifically utilized RT4 cells, a well-characterized, non-invasive bladder cancer model, to investigate the impact of proTAME, cisplatin, and gemcitabine on MMP2/MMP9-mediated migration, establishing a foundation for subsequent research using more aggressive bladder cancer models. Although this investigation relied on 2D cell culture to characterize molecular changes, future studies will employ 3D spheroid models to better replicate the tumor microenvironment and confirm these findings in a more physiologically relevant context. Additionally, future research will incorporate protein-level assays and functional invasion assays to validate the effects of these agents on MMP activity and cellular invasiveness. Furthermore, the potential mechanistic link between APC/C inhibition and MMP regulation through CDC20/CDH1 modulation, as suggested by preliminary docking analyses, warrants biochemical and functional validation using alternative APC/C inhibitors in subsequent studies.

## 5. Conclusions

In conclusion, the present study demonstrates that proTAME and its combination therapies with cisplatin and gemcitabine are associated with reduced cell migration and downregulated MMP2 expression in bladder cancer cells, although further studies are necessary to determine whether these effects reflect anti-migratory activity or indirect influences on cell viability and stress responses. These findings suggest that proTAME may enhance the effectiveness of standard chemotherapy through potential modulation of MMP-mediated pathways involved in tumor invasion and metastasis. Given the observed effects of proTAME on migration pathways and MMP regulation, it may also hold promise as a repurposed agent for conditions involving aberrant ECM remodeling and cell migration. Future research exploring these applications, along with further validation in advanced preclinical models, could expand the therapeutic scope of proTAME beyond bladder cancer. The repurposed potential and notable combination effects of proTAME highlight it as a promising candidate for further investigation in cancer therapy and other biomedical applications, contingent upon validation in additional functional studies.

## Figures and Tables

**Table 1:** Grid box parameters for molecular docking simulations.

Protein	Center at (X, Y, Z)	Dimension (Å)
MMP2	X: 8.587, Y: 20.237, Z: 12.663	40 Å × 40 Å × 40 Å
MMP9	X: 54.532, Y: 21.247, Z: 129.543	40 Å × 40 Å × 40 Å

**Table 2:** Summary of molecular docking analysis results for proTAME, gemcitabine, cisplatin, and I52 with MMP2. The table presents the minimum binding affinity (kcal/mol), number of conventional hydrogen bonds, average hydrogen bond distances (Å), and key structural features contributing to the interaction stability for each ligand. These results provide a comparative overview of the binding characteristics and potential inhibitory effectiveness of the analyzed ligands.

Protein/Ligand	Minimum Binding Affinity (Kcal/mol)	No. of Conventional Hydrogen Bonds	Hydrogen Bond Distance (Å)	Key Structural Amino Acid Residues
MMP2–proTAME	−9.2	3	2.74	GLY73
			2.79	HIS85
			2.16	TYR142
MMP2–gemcitabine	−7.0	3	2.02	LEU83
			2.54	ALA84
			3.15	VAL117
MMP2–cisplatin	−3.2	2	2.43	ASP101
			1.98	ASP101
MMP2–I52	−8.1	4	2.51	LEU82
			2.10	LEU83
			3.02	ALA84
			2.14	ALA84

**Table 3:** Summary of molecular docking analysis results for proTAME, gemcitabine, cisplatin, and NFH with MMP9. The table presents the minimum binding affinity (kcal/mol), number of conventional hydrogen bonds, average hydrogen bond distances (Å), and key structural features contributing to the interaction stability for each ligand. These results provide a comparative overview of the binding characteristics and potential inhibitory effectiveness of the analyzed ligands.

Protein/Ligand	Minimum Binding Affinity (Kcal/mol)	No. of Conventional Hydrogen Bonds	Hydrogen Bond Distance (Å)	Key Structural Amino Acid Residues
MMP9–proTAME	−8.7	3	3.07	GLY186
			1.83	LEU188
			2.62	TYR423
MMP9–gemcitabine	−8.6	1	3.21	GLU416
MMP9–cisplatin	−3.7	6	2.20	ARG424
			2.12	MET422
			2.78	LEU418
			2.86	TYR420
			2.64	TYR420
			2.88	LEU418
MMP9–NFH	−6.2	4	1.83	LEU188
			2.51	TYR423
			2.30	PRO421
			2.06	GLY186

## Data Availability

The data presented in this study are available on request from the corresponding authors.
